# Magnetic Resonance Imaging for Surveillance of Hepatocellular Carcinoma: A Systematic Review and Meta-Analysis

**DOI:** 10.3390/diagnostics11091665

**Published:** 2021-09-12

**Authors:** Dong Hwan Kim, Sang Hyun Choi, Ju Hyun Shim, So Yeon Kim, Seung Soo Lee, Jae Ho Byun, Kyung Won Kim, Joon-Il Choi

**Affiliations:** 1 Department of Radiology, Seoul St. Mary’s Hospital, College of Medicine, The Catholic University of Korea, Seoul 06591, Korea; kimdh@catholic.ac.kr (D.H.K.); dumkycji@gmail.com (J.-I.C.); 2 Department of Radiology and Research Institute of Radiology, University of Ulsan College of Medicine, Asan Medical Center, Seoul 05505, Korea; sykim.radiology@gmail.com (S.Y.K.); seungsoolee@amc.seoul.kr (S.S.L.); jhbyun@amc.seoul.kr (J.H.B.); medimash@gmail.com (K.W.K.); 3 Department of Gastroenterology, University of Ulsan College of Medicine, Asan Medical Center, Seoul 05505, Korea; s5854@amc.seoul.kr

**Keywords:** liver, hepatocellular carcinoma, surveillance, magnetic resonance imaging, systematic review, meta-analysis

## Abstract

Our meta-analysis aimed to evaluate the diagnostic performance of surveillance magnetic resonance imaging (sMRI) for detecting hepatocellular carcinoma (HCC), and to compare the diagnostic performance of sMRI between different protocols. Original articles about the diagnostic accuracy of sMRI for detecting HCC were found in major databases. The meta-analytic pooled sensitivity and specificity of sMRI for detecting HCC were determined using a bivariate random effects model. The pooled sensitivity and specificity of full MRI and abbreviated MRI protocols were compared using bivariate meta-regression. In the total seven included studies (1830 patients), the pooled sensitivity of sMRI for any-stage HCC and very early-stage HCC were 85% (95% confidence interval, 79–90%; *I*^2^ = 0%) and 77% (66–85%; *I*^2^ = 32%), respectively. The pooled specificity for any-stage HCC and very early-stage HCC were 94% (90–97%; *I*^2^ = 94%) and 94% (88–97%; *I*^2^ = 96%), respectively. The pooled sensitivity and specificity of abbreviated MRI protocols were 87% (80–94%) and 94% (90–98%), values that were comparable with those of full MRI protocols (84% [76–91%] and 94% [89–99%]; *p* = 0.83). In conclusion, sMRI had good sensitivity for detecting HCC, particularly very early-stage HCC. Abbreviated MRI protocols for HCC surveillance had comparable diagnostic performance to full MRI protocols.

## 1. Introduction

Hepatocellular carcinoma (HCC) is the fifth most frequent malignancy and the fourth leading cause of cancer-related deaths [1,2]. Although the overall prognosis for patients with advanced HCC is extremely poor, patients who are diagnosed in the early-stages of HCC are eligible for curative treatments such as surgical resection, local ablation, or transplantation, and can be expected to have improved probability of survival [3,4,5]. Therefore, the detection of early-stage HCC by surveillance is important in prolonging survival and reducing mortality of patients at risk for HCC [6,7].

Currently, the American Association for the Study of Liver Disease (AASLD) and the European Association for the Study of the Liver recommend ultrasound (US) examinations around 6 month intervals for patients in at-risk populations, especially with cirrhosis [3,4]. Although US is the most cost-effective surveillance modality [8,9], the performance of US for detecting early-stage HCC is limited, with a pooled sensitivity of 47% being reported in a recent meta-analysis [10]. Given this limitation of US, the need for alternative imaging modalities for HCC surveillance is increasing, with these including magnetic resonance imaging (MRI) and computed tomography (CT).

MRI has been widely used in the diagnosis of HCC. Although MRI has better performance for detecting and characterizing liver lesions than US or CT [11,12], MRI is suboptimal for HCC surveillance because of its cost, the long examination time, and its complexity [13]. Recently, abbreviated MRI protocols using a reduced number of sequences were introduced, and the use of abbreviated MRI protocols for HCC surveillance is drawing increasing attention [13,14,15,16]. However, the reported results show variation between studies [13,17] and are limited by low numbers of subjects [13,15,16]. Considering the potential benefit of MRI in HCC surveillance, it might be important to determine the diagnostic performance of MRI for HCC surveillance, especially abbreviated MRI protocols. Therefore, this meta-analysis aimed to evaluate the diagnostic performance of surveillance MRI (sMRI) for the detection of HCC, and to compare the diagnostic performance of sMRI according to different MRI protocols.

## 2. Materials and Methods

This systematic review and meta-analysis was conducted according to the guidance of the Preferred Reporting Items for Systematic Reviews and Meta-Analyses (PRISMA) guideline [18] and was prospectively registered in PROSPERO (ID: CRD 42020185118).

### 2.1. Eligibility Criteria

Articles were reviewed with respect to eligibility according to the following criteria: (i) population, patients at risk of HCC; (ii) index test, liver MRI for HCC surveillance; (iii) reference standard, pathological diagnosis, or imaging follow-up; (iv) outcomes, sensitivity and specificity of MRI for the detection of HCC. Patients at risk for HCC were defined as those with cirrhosis or chronic liver disease. In this meta-analysis, we considered a repeated test at a regular interval over time for the detection of previously undiagnosed lesions as surveillance [10], and studies performing evaluations for diagnostic purposes instead of surveillance were not included in this analysis. The exclusion criteria were as follows: (i) review articles, editorials, case reports, scientific conference abstracts/proceedings, or non-human research; (ii) studies with overlapping patient cohorts and data; (iii) studies that were not within the field of interest of this study; and (iv) studies without sufficient details to construct a diagnostic 2-by-2 table of the imaging results and reference standard findings. Articles were first screened by their titles and abstracts, with full-text reviews then being performed after the selection of potentially eligible abstracts. Both steps were performed by 2 independent reviewers (with 8 and 3 years of experience in meta-analysis). The 2 reviewers excluded only those articles that were clearly ineligible. Other articles with any degree of ambiguity or that generated differences between the 2 reviewers were re-evaluated at a consensus meeting with a third invited reviewer.

### 2.2. Search

Thorough searches of MEDLINE, EMBASE, and Cochrane databases were performed to find original articles about the diagnostic performance of sMRI for detecting HCC. A manual evaluation of the identified articles was conducted to find eligible articles and to narrow down the number of relevant articles. The search terms included “hepatocellular carcinoma,” “MRI,” “surveillance,” and “screen” (Table S1). The beginning date for the search was set as 1 January 2000, and the literature search was updated until 22 April 2020. 

### 2.3. Data Items

The two reviewers independently reviewed and extracted the following data: (i) study characteristics (authors, year of publication, country, institution, and study design); (ii) subject characteristics (number of subjects, age, sex, underlying liver disease, number of lesions, lesion size, and the prevalence of HCC); (iii) MRI techniques (scanner field strength, type of contrast agents, and MRI protocols used); (iv) reference standards; and (v) outcomes (the accuracy of MRI for the detection of HCC). In the case of all discrepancies, a consensus meeting in the presence of a third reviewer was performed. 

### 2.4. Risk of Bias in Individual Studies

The Quality Assessment of Diagnostic Accuracy Studies (QUADAS-2) criteria [19] were used to assess the quality of the selected articles. The QUADAS-2 tool assesses study quality in the 4 different domains of patient selection, index test, reference standard, and flow of patients through the study and timing of the index test and reference standard. 

### 2.5. Summary Measures

To evaluate the diagnostic performance of sMRI for detecting any-stage, early-stage, or very early-stage HCC, the per-patient sensitivity and specificity with 95% CIs were calculated for each individual study. The Barcelona Clinic Liver Cancer (BCLC) staging system was used to determine HCC staging, i.e., early-stage = BCLC stage A and very early-stage = BCLC stage 0 [20]. 

### 2.6. Synthesis of Results

The meta-analytic pooled sensitivity and specificity were determined using a bivariate random-effects model. The summary receiver operating characteristic curve was provided using the hierarchical summary receiver operating characteristics (HSROC) model. The study heterogeneity was evaluated using Higgins *I*^2^ statistic, with an *I*^2^ > 50% being considered to indicate substantial heterogeneity. When heterogeneity was noted, the threshold effect was evaluated using the coupled forest plots and the Spearman correlation coefficient between sensitivity and false-positive rate. A correlation coefficient > 0.6 was considered to indicate a considerable threshold effect. Subgroup analyses were conducted on prospective studies and studies that exclusively included patients with cirrhosis. To evaluate the performance of MRI in combination with alpha-fetoprotein (AFP), the meta-analytic summary area under the receiver operating characteristics curve (AUROC) and its 95% CI were calculated for the available studies using a random-effect model. The diagnostic performance for HCC detection was compared between studies with full MRI protocols and those with abbreviated MRI protocols using joint-model bivariate meta-regression.

Sensitivity analysis and meta-regression analysis were additionally performed. The meta-regression analysis considered the following covariates: (i) proportion of HCC less than 2 cm (<50% or not reported, vs. >50%); (ii) the most common etiology of the underlying liver disease (hepatitis C vs. hepatitis B); (iii) HCC prevalence (<10% vs. >10%); (iv) study location (Western vs. Eastern); (v) study period (<2005 vs. ≥2005); (vi) MRI magnet field strength (3.0T vs. 1.5T, 1.5/3.0T, or not reported); (vii) MRI contrast agent (hepatocyte-specific contrast agent vs. extracellular contrast agent); (viii) reference standard for HCC (imaging only vs. pathology or imaging); (ix) reference standard for non-HCC (explantation only vs. imaging follow-up); and (x) follow-up period (mean period < 6 months vs. mean period ≥ 6 months).

Deeks’ funnel plot and Deeks’ asymmetry test were used to evaluate publication bias. All statistical analysis was performed using the Stata version 16.0 (StataCorp LP, College Station, TX, USA).

## 3. Results

### 3.1. Literature Search

**Figure 1:**
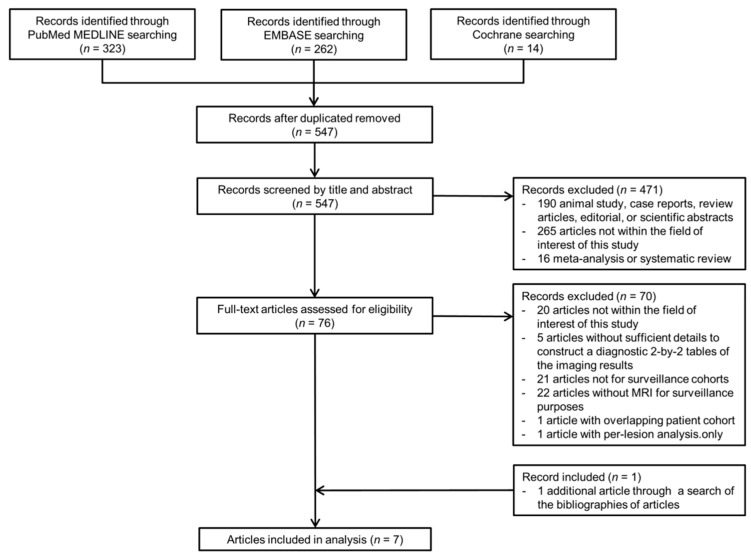
PRISMA flow diagram.

After removing duplicate articles, 547 articles were screened, and 471 articles were excluded in the first screening on the basis of their titles and abstracts (Figure 1). A total of 70 articles were additionally excluded in the second screening on the basis of a full-text review. A search of the bibliographies of the remaining articles yielded one additional eligible article. Finally, seven articles were included in this systematic review and meta-analysis [13,14,15,16,17,21,22]. 

The study characteristics of the seven included articles are summarized in Table 1. In a total of 1830 subjects who underwent surveillance, 195 developed HCC. Of the seven included studies, three were prospective (71 HCCs in 909 patients) [15,17,21] and three exclusively included patients with cirrhosis (100 HCCs in 1011 patients) [17,21,22]. The most common etiology of underlying liver disease was hepatitis C in three studies [13,14,17] and hepatitis B in four studies [15,16,21,22]. Two studies used only 3.0T MRI [16,22], and three used only a hepatocyte-specific contrast agent [13,14,21]. In all but one study [16], the final diagnosis of HCC, non-HCC malignancy, and benignity was determined by a combination of pathological diagnosis and imaging follow-up.

### 3.2. Study Quality Assessment

**Figure 2:**
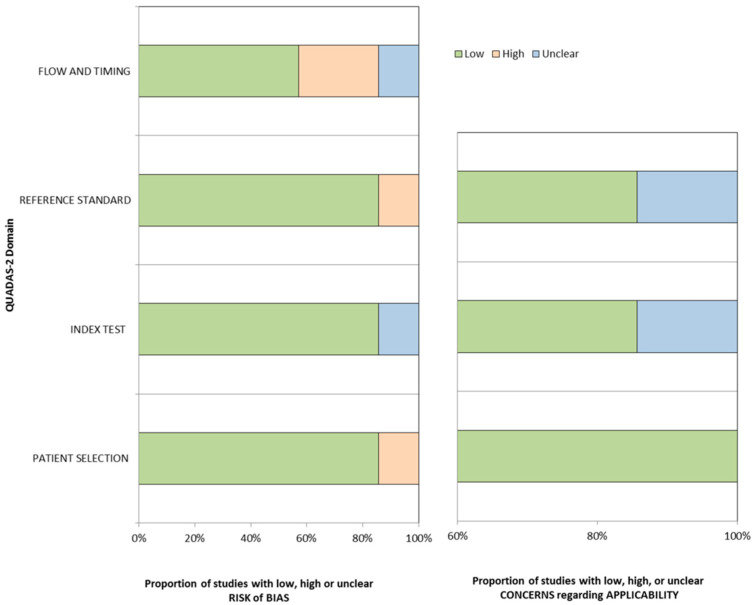
Results of study quality assessments according to Quality Assessment Tool for Diagnostic Accuracy-2 criteria.

Figure 2 shows the results of quality assessment for the seven included articles. In the patient selection domain, one study had a high risk of bias because it included patients listed for liver transplantation, potentially introducing a selection bias [17]. In the index test domain, the blindness between the index test results and the reference standard results were unclear in all but one study [22]. Six studies used pathologic diagnosis with the imaging test as a reference standard, but one study used only the imaging test, causing a lack of independence between surveillance and reference tests, which could potentially overestimate the performance of the surveillance test [16]. The interval between the index test and reference standard was approximately 1 year in two studies [13,14], resulting in a high risk of bias in the flow and timing domain.

### 3.3. Diagnostic Performance of sMRI for the Detection of HCC

**Figure 3:**
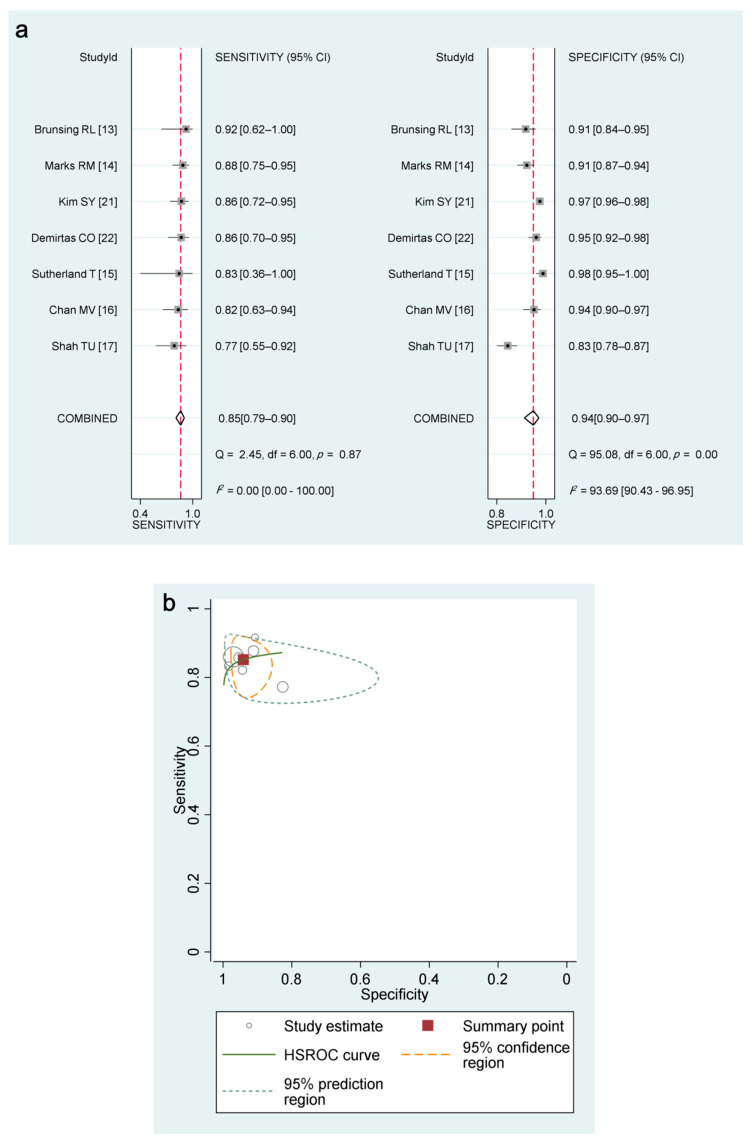
Coupled forest plots and HSROC curve for sMRI. (**a**) The quadrilateral of the combined row in the coupled forest plots represents the pooled estimation and its 95% confidence interval. (**b**) The large difference between 95% confidence region and 95% prediction region in HSROC curve indicates the presence of substantial study heterogeneity.

The seven studies (1830 patients) were available for the performance of MRI for detecting any-stage HCC. The pooled sensitivity and specificity of sMRI for any-stage HCC were 85% (95% CI, 79–90%; *I*^2^ = 0%) and 94% (95% CI, 90–97%; *I*^2^ = 94%), respectively (Table 2, Figure 3).

**Figure 4:**
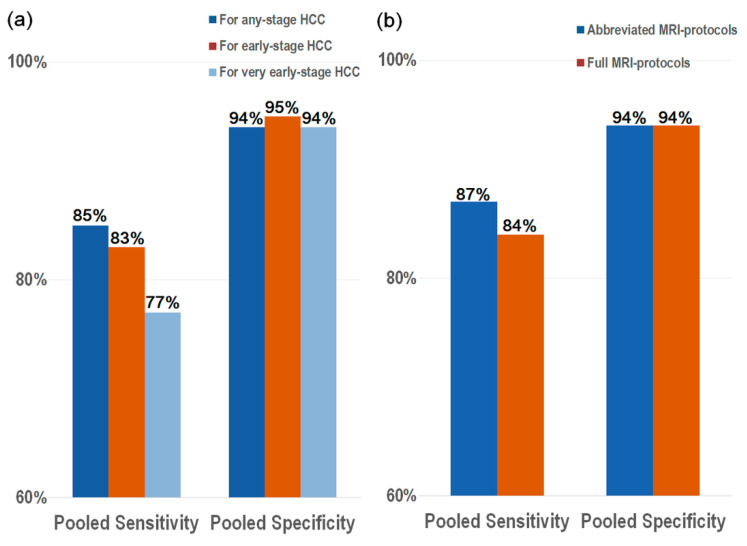
Performance of surveillance MRI for the detection of HCC according to (**a**) HCC staging and (**b**) MRI protocol. The performance of the abbreviated MRI protocols for detecting any-stage HCC was comparable with those of the full MRI protocols (*p* = 0.83).

Five studies were available for the diagnostic performance of MRI for detecting early-stage HCC (107 HCCs in 1344 patients) [13,15,17,21,22]. The pooled sensitivity specificity was 83% (95% CI, 74–89%; *I*^2^ = 0%) and 95% (95% CI, 89–97; *I*^2^ = 96%), respectively (Table 2 and Figure 4). Four studies were available for the diagnostic performance of MRI for detecting very early-stage HCC (86 HCCs in 1199 patients) [16,17,21,22], with the pooled sensitivity and specificity for this being 77% (95% CI, 66–85%; *I*^2^ = 32%) and 94% (95% CI, 88–97%; *I*^2^ = 96%), respectively (Table 2 and Figure 4). There was no significant threshold effect between sensitivity and specificity (rho ≤ 0.0, *p* ≥ 0.20).

In the subgroup analysis of prospective studies, the pooled sensitivity and specificity of sMRI for any-stage HCC were 83% (95% CI, 74–92%) and 95% (95% CI, 91–99%), respectively, whereas in the subgroup analysis for studies that exclusively included patients with cirrhosis, those were 84% (95% CI, 76–91%) and 94% (95% CI, 89–99%), respectively.

Two studies compared the performance of sMRI with or without AFP measurement for the detection of any-stage HCC [21,22]. The pooled AUROC of sMRI alone was similar to that of sMRI in conjunction with AFP measurement (0.92 [95% CI, 0.88–0.96]; *I*^2^ = 0% vs. 0.93 [95% CI, 0.89–0.97]; *I*^2^ = 0%).

### 3.4. Full MRI Protocols vs. Abbreviated MRI Protocols

In the seven studies for any-stage HCC, three (*n* = 1011) used full MRI protocols, and four (*n* = 819) used abbreviated MRI protocols (Figure 4). The abbreviated MRI protocols consisted of various combinations of imaging sequences without dynamic contrast-enhanced imaging, but including T1-weighted imaging, T2-weighed imaging (T2WI), diffusion-weighted imaging (DWI), and hepatobiliary-phase imaging. The pooled sensitivity and specificity of the abbreviated MRI protocols for detecting any-stage HCC were 87% (95% CI, 80–94%) and 94% (95% CI, 90–98%), values that were comparable with those of the full MRI protocol (84% [95% CI, 76–91%] and 94% [95% CI, 89–99%]; *p* = 0.83; Figure 4). The pooled sensitivity and specificity of the abbreviated MRI protocols for early-stage HCC were 85% (95% CI, 66–100%) and 96% (95% CI, 91–100%), which were comparable with those of the full MRI protocols (83% [95% CI, 74–91%] and 94% [95% CI, 88–99%]; *p* = 0.84; Table S2). Of the four studies for very early-stage HCC, three used full MRI protocols, and one used an abbreviated MRI protocol. The abbreviated MRI protocol had lower sensitivity for detecting very early-stage HCC than the full MRI protocols, but the difference was not statistically significant (59% [95% CI, 34–83%] vs. 81% [95% CI, 71–91%], *p* = 0.16). Both abbreviated and full MRI protocols showed similar specificity (95% [95% CI, 87–100%] and 94% [95% CI, 89–99%]; Table S2).

In the subgroup analysis of prospective studies, the pooled sensitivity and specificity were 83% (95% CI, 42–99%) and 98% (95% CI, 95–100%), respectively, for abbreviated MRI protocols, and 82% (95% CI, 66–92%) and 93% (95% CI, 65–99%), respectively, for full MRI protocols. It was not possible to compare full and abbreviated MRI protocols for the studies that exclusively included patients with cirrhosis because all three such studies used full MRI protocols.

### 3.5. Sensitivity Analysis and Meta-Regression Analysis

The study by Shah et al. [17] (by far the earliest study) reported a relatively low sensitivity and specificity in comparison with the other included studies, and the study heterogeneity affecting specificity was lower (*I*^2^ = 81%) after this study [17] was excluded (Table S3). However, the pooled sensitivity and specificity after excluding the study by Shah et al. [17] were 86% and 95%, respectively, which were similar to the values for all seven included studies. 

The most common etiology of underlying liver disease and the type of MRI contrast agent were significant factors associated with study heterogeneity (*p* ≤ 0.01) (Table S4). Studies in which hepatitis B was the most common etiology of liver disease showed a higher specificity than those in which it was hepatitis C (97% vs. 88%). Studies using hepatocyte-specific contrast agents showed both higher sensitivity (87% vs. 82%) and specificity (94% vs. 91%) than those with extracellular contrast agents. The study period and reference standard for non-HCC showed a borderline statistical significance (*p* = 0.05).

Publication bias did not significantly exist among the included studies (*p* = 0.93, Figure S1).

## 4. Discussion

This study found that sMRI demonstrated a good diagnostic performance for both any-stage HCC and very early-stage HCC, with pooled sensitivities of 85% and 77%, respectively, and pooled specificities of 94% for both. In addition, the performance of abbreviated MRI protocols was comparable to that of full MRI protocols, with sensitivities of 87% vs. 84%, respectively, and specificities of 94% vs. 94% (*p* = 0.83). Because we included only studies that evaluated the diagnostic performance of MRI for surveillance purposes in the surveillance cohorts, our meta-analysis would be useful to apply to the clinical practice of HCC surveillance.

**Figure 5:**
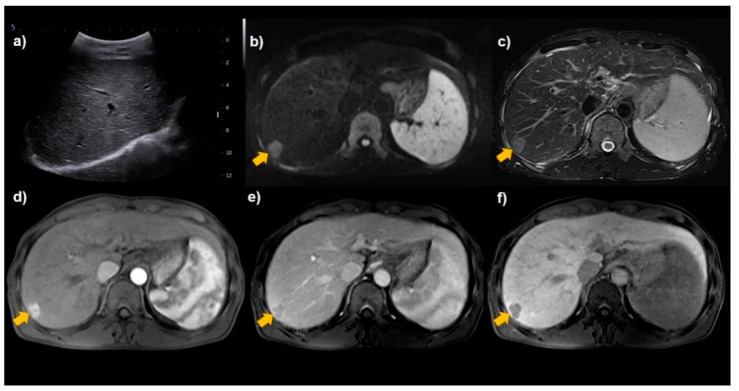
A 60-year-old male patient with chronic hepatitis B. (**a**) No focal hepatic lesion was found in surveillance ultrasonography due to poor sonic window. (**b**–**f**) Surveillance MRI detected a 2.0 cm nodule in segment VII (arrows). MRI shows diffusion restriction (**b**) and mild-to-moderate T2 hyperintensity (**c**) with arterial-phase hyperenhancement (**d**), portal venous washout (**e**), and hepatobiliary-phase hypointensity (**f**), suggesting HCC.

sMRI had a high detection rate for any-stage HCC, early-stage HCC, and very early-stage HCC. Compared with the performance of US reported in a previous meta-analysis [10], MRI had similar sensitivity for any-stage HCC (MRI 85% vs. US 84%). However, the sensitivity of MRI for both ‘early-stage HCC’ and ‘very early-stage HCC’ in our meta-analysis was higher than that of US for ‘early-stage HCC’ in the previous meta-analysis (83% vs. 47% and 77% vs. 47%). Although US has been considered as a standard surveillance test for HCC because of its excellent accessibility and non-invasiveness [3,4,5,23,24], some factors, including macronodular cirrhosis, severe obesity, and a subcapsular tumor location, may lead to surveillance failure, and the sensitivity of US may be particularly impaired in patients with advanced liver cirrhosis due to the heterogeneity of the liver parenchyma and progressed parenchymal distortion (Figure 5) [25,26,27]. Likewise, a recent prospective surveillance study of patients with cirrhosis [21] found that sMRI had higher sensitivity than US for detecting very early-stage HCC (84.8% vs. 27.3%). Although MRI has limitations to clearly differentiate between tumor progression and necrosis, sMRI might be clinically useful to detect early-stage HCC in patients at risk of HCC, considering the fact that the goal of HCC surveillance is to detect disease early to initiate potentially curative interventions and reduce overall morbidity and mortality [28].

Despite its high detection rate for HCC, MRI has several limitations, including its high cost, long scan time, and complexity, which can hinder widespread adoption in all at-risk patients. In addition, MRI requires a scanning room with an effective radio-frequency shield and takes more preparation time, i.e., the time for entering and exiting the scanning room or for preparing contrast injection. Therefore, sMRI in all at-risk patients might not be cost-effective, and might be better for patients who have a sufficiently high risk of HCC [29]. To overcome these limitations, abbreviated MRI protocols using a reduced number of sequences have been introduced. In this meta-analysis, the performance of abbreviated MRI protocols for detecting any-stage HCC was found to be comparable to that of full MRI protocols. Notably, in the case of non-enhanced abbreviated MRI protocols consisting of DWI or T2WI, additional advantages can be achieved through lowering the cost, decreasing the time required for preparing contrast injection, and increasing safety by not using contrast agents [30]. Considering the 30–49% cost saving of abbreviated MRI protocols in comparison with full MRI protocols [31], and that abbreviated MRI for intermediate- to high- risk patients with cirrhosis and US for lower-risk patients is cost-effective [32], abbreviated MRI protocols may be clinically useful for HCC surveillance.

Study heterogeneity was significantly associated with the type of MRI contrast agent and the etiology of underlying liver disease. MRI using hepatocyte-specific contrast agent has been increasingly used because of its improved sensitivity for detecting small HCCs [33]. Hepatocyte-specific contrast agent such as gadoxetate can act as an extracellular contrast agent to evaluate hemodynamic change and at later times enhances hepatocytes via the organic anion transporting polypeptide transporter expressed at the sinusoidal membrane [34]. The better performance of MRI using hepatocyte-specific contrast agent might be explained by the higher lesion conspicuity and lesion-to-liver contrast during the hepatobiliary phase of hepatocyte-specific contrast in comparison with the equilibrium phase of extracellular contrast [34]. Although sensitivity is generally considered to be of utmost importance in surveillance modalities for optimizing early HCC detection, balancing specificity to minimize surveillance-related harms is also a crucial issue. Especially in a transplantation scenario, organ shortage remains a major limitation, and achieving high specificity is important for maximizing organ utilization. In our study results, the specificity of sMRI for the detection of HCC was very high (94–95%) regardless of HCC staging or MRI protocol. In addition, the performance of sMRI might differ according to the underlying liver disease. Considering the fact that cirrhosis usually accompanies chronic hepatitis C infection and precedes hepatocarcinogenesis [35], a lower specificity (i.e., higher false-positive rate) might be expected in hepatitis C.

Our study has several study limitations. First, four of the seven included studies were retrospective by design. In addition, the small number of included studies may produce a lower statistical power. However, as we exclusively included studies performing evaluations for surveillance purposes, the results of our study would be more reliable to determine the diagnostic performance of sMRI. Second, substantial study heterogeneity was noted for the specificity. In the meta-regression analysis, the reference standard for HCC was not associated with study heterogeneity, but that for non-HCC showed a borderline statistical significance. The fact that the reference standard for the diagnosis of HCC was similar throughout the studies (i.e., pathology or multiphasic CT/MRI by the AASLD HCC practice guideline) but the reference standard for the absence of HCC was variable (i.e., follow-up US, CT, MRI vs. explantation only) might explain why significant study heterogeneity was noted for specificity, but not sensitivity. To overcome the heterogeneity, additional analyses including subgroup analysis, sensitivity analysis, and meta-regression analysis were performed. The sensitivity analysis showed lower study heterogeneity was decreased after excluding the study by Shah et al. [17], and study period showed a borderline statistical significance in the meta-regression analysis. These results might be associated with the MRI techniques, as Shah et al. [17] used thicker image slices than the other studies (7–10 mm vs. 3–5 mm). Third, the incidence of HCC in this meta-analysis might be lower than in clinical practice because four studies included at-risk patients rather than patients with cirrhosis or chronic hepatitis B. Third, this meta-analysis did not include scientific abstracts or conference proceedings.

## 5. Conclusions

In conclusion, sMRI had good sensitivity for detecting HCC, particularly very early-stage HCC. In HCC surveillance, abbreviated MRI protocols had comparable diagnostic performance to full MRI protocols. Therefore, sMRI, including abbreviated MRI protocols, might be clinically useful as a surveillance strategy for patients at risk of HCC.

## Figures and Tables

**Table 1:** Study characteristics of the seven articles.

Author (Year of Publication)	Study Design	Study Location (Period)	No. of Patients (% Male)	Percentage of Cirrhotic Patients (%)	Most Common Etiology of Liver Disease (%)	No. of Patients with HCC (% Early HCC)	Patient Age, Year *	MRI Magnet	MRI Protocols	MRI Contrast Agent	Reference Standards for HCC (%)	Surveillance Interval (f/u Period), Month
Shah TU (2006) [17]	Prospective	United States (2001–2004)	310 (66.5)	100	Hepatitis C (25.5)	22 (100)	52.4, mean	1.5-T	T1WI, T2WI, DCE	Extracellular contrast agent	Pathology (42.9) or multiphase MRI (57.1)	3–12 (f/u: mean, 22.1)
Marks RM (2015) [14]	Retrospective	United States (2008–2012)	298 (56.4)	NR	Hepatitis C (50.7)	49 (NR)	55.9 ± 10.9	1.5 or 3.0-T	T2WI, HBP, DWI	Hepatocyte-specific contrast agent	Pathology (NR), multiphase CT or MRI (NR)	NR (f/u: range, 6–13)
Kim SY (2017) [21]	Prospective	Korea (2011–2014)	407 (56.5)	100	Hepatitis B (70.8)	43 (97.7)	56 (52–62), median (IQR)	1.5-T	T2WI, DWI, Dual-GRE, DCE, HBP	Hepatocyte-specific contrast agent	Pathology (9.3) or multiphase CT (90.7)	6 (f/u: median, 18)
Sutherland T (2017) [15]	Prospective	Australia (NR)	192 (72.4)	NR	Hepatitis B (56.3)	6 (12.2)	58 (22–80), mean (range)	NR	DWI	NA	Pathology (NR), multiphase CT or MRI (NR)	NR (f/u: > 6)
Brunsing RL (2019) [13]	Retrospective	United States (2014–2016)	141 (54.6)	92.9	Hepatitis C (37.9)	12 (66.7)	59.1 ± 11.5	1.5 or 3.0-T	T2WI, HBP, DWI	Hepatocyte-specific contrast agent	Pathology (NR), multiphase CT or MRI (NR)	5.0–8.8 (f/u: range, 8.8–26)
Chan MV (2019) [16]	Retrospective	Australia (2015–2018)	188 (49.5)	23.4	Hepatitis B (14.9)	28 (NR)	63 ± 13	3.0-T	T2WI, DWI, Dual-GRE	NA	Multiphase MRI (100)	6 (f/u: NR)
Demirtas CO (2020) [22]	Retrospective	Turkey (2008–2017)	294 (37.1)	100	Hepatitis B (41.5)	35 (85.7)	60 (29–86), median (IQR)	3.0-T	NR ^†^	Extracellular contrast agent	Pathology (NR), multiphase CT or MRI (NR)	12 (f/u: mean, 40.3)

* Unless otherwise indicated, data are mean ± standard deviation. ^†^ Although the MRI sequences used were not explicitly mentioned, it was judged by consensus among the reviewers that a full MRI protocol was used in this study. MRI, magnetic resonance imaging; HCC, hepatocellular carcinoma; IQR, interquartile range; T1WI, T1-weighted images; T2WI, T2-weighted images; DCE, dynamic contrast enhancement; HBP, hepatobiliary phase; DWI, diffusion-weighted images; Dual-GRE, T1-weighted dual gradient-echo out-of-phase images and in-phase; NR, not reported; NA, not applicable; CT, computed tomography; f/u, follow-up.

**Table 2:** Diagnostic performance of sMRI for any-stage HCC, early-stage HCC, and very early-stage HCC.

Any-Stage HCC			Early-Stage HCC			Very Early-Stage HCC		
Author (Year)	Sensitivity (95% CI)	Specificity (95% CI)	Author	Sensitivity (95% CI)	Specificity (95% CI)	Author	Sensitivity (95% CI)	Specificity (95% CI)
Shah TU (2006) [17]	77% (55, 92)	83% (78, 87)	Shah TU [17]	77% (55, 92)	83% (78, 87)	Shah TU [17]	77% (55, 92)	83% (78, 87)
Marks RM (2015) [14]	88% (75, 95)	91% (87, 94)	Kim SY [21]	86% (71, 95)	97% (96, 98)	Kim SY [21]	84% (67, 95)	97% (96, 98)
Kim SY (2017) [21]	86% (71, 94)	97% (96, 98)	Sutherland T [15]	80% (28, 99)	98% (95, 100)	Chan MV [16]	59% (33, 82)	95% (90, 98)
Sutherland T (2017) [15]	83% (42, 99)	98% (95, 100)	Brunsing RL [13]	88% (47, 100)	91% (85, 95)	Demirtas CO [22]	80% (52, 96)	96% (93, 98)
Brunsing RL (2019) [13]	92% (60, 100)	91% (84, 95)	Demirtas CO [22]	83% (65, 94)	95% (92, 98)			
Chan MV (2019) [16]	82% (62, 93)	94% (89, 97)						
Demirtas CO (2020) [22]	86% (69, 95)	95% (92, 98)						
Meta-analytic pooled estimations	85% (79, 90)	94% (90, 97)		83% (74, 89)	95% (89, 97)		77% (66, 85)	94% (88, 97)

CI, confidence interval; HCC, hepatocellular carcinoma; MRI, magnetic resonance imaging.

## Data Availability

All data accessed and analyzed in this study are available in the article and its Supplementary Materials.

## Supplementary Materials

The following are available online at https://www.mdpi.com/article/10.3390/diagnostics11091665/s1, Supplementary Table S1: Search queries, Supplementary Table S2: performance of abbreviated MRI-protocols and full MRI-protocols for the detection of early-stage HCC and very early-stage HCC, Supplementary Table S3: results of sensitivity analysis of surveillance MRI for the detection of hepatocellular carcinoma, Supplementary Table S4: results of meta-regression analysis of surveillance MRI for the detection of hepatocellular carcinoma, Supplementary Figure S1: Deeks’ funnel plot to evaluate publication bias of surveillance MRI.
