# Supplementary material for: Magnetic Resonance Imaging for Surveillance of Hepatocellular Carcinoma: A Systematic Review and Meta-Analysis
Source: Diagnostics (Basel). 2021 Sep 12;11(9):1665. doi: 10.3390/diagnostics11091665 (PMC8469328; doi:10.3390/diagnostics11091665)

Supplementary Table S1. Search queries

No.	Search queries for MEDLINE
#1	"Carcinoma, Hepatocellular"[Mesh] OR "Liver Neoplasms"[Mesh:NoExp]
#2	(liver*[TW] OR hepatic*[TW] OR Hepato*[TW]) AND (carcinoma*[TW] OR cancer[TW] OR cancers[TW] OR tumor[TW] OR neoplas*[TW] OR malignan*[TW])
#3	hepatocarcinoma*[TW] OR hepatoma*[TW] OR "liver carcinoma"[TW] OR HCC[TW]
#4	#1 OR #2 OR #3
#5	"Magnetic Resonance Imaging"[Mesh]
#6	Magnetic-Resonanc*[TW] OR "MR"[TI] OR "MRI"[TW] OR "diffusion-weighted"[TW]
#7	#5 OR #6
#8	abbreviat*[TW] OR surveillanc*[TW] OR screen*[TW]
#9	"Early Detection of Cancer"[Mesh] OR "mass screening"[MeSH] OR "Epidemiological Monitoring"[Mesh]
#10	#8 OR #9
#11	"Predictive Value of Tests"[Mesh] OR "Sensitivity and Specificity"[Mesh]
#12	predictive value*[TW] OR detection rate*[TW] OR "False Negative"[TW] OR "False positive"[TW] OR "True Negative"[TW] OR "True positive"[TW] OR "PPV"[TW] OR "NPV"[TW] OR Sensitivit*[TW] OR Specificit*[TW]
#13	"Reproducibility of Results"[Mesh]
#14	accurac*[TW] OR Validit*[TW]
#15	#11 OR #12 OR #13 OR #14
#16	#4 AND #7 AND #10 AND #15
#17	#16 AND ("2000/01/01"[PDAT] : "3000/12/31"[PDAT]) AND (English[Lang])
No.	Search queries for EMBASE
#1	'liver cell carcinoma'/exp OR 'liver cancer'/de
#2	((liver* OR hepatic* OR Hepato*) NEAR/6 (Carcinoma* OR Cancer* OR tumor* OR Neoplas* OR malignan*)):ab,ti,kw
#3	(hepatocarcinoma* OR hepatoma* OR 'liver carcinoma' OR HCC):ab,ti,kw
#4	#1 OR #2 OR #3
#5	'nuclear magnetic resonance imaging'/exp OR 'MR':ti OR 'MRI':ab,ti,kw

#6	(Magnetic-Resonanc* OR 'diffusion-weighted'):ab,ti,kw
#7	#5 OR #6
#8	(abbreviat* OR surveillanc* OR screen*):ab,ti,kw
#9	'early cancer diagnosis'/exp OR 'cancer screening'/exp OR 'disease surveillance'/exp
#10	#8 OR #9
#11	'diagnostic accuracy'/exp OR 'predictive value'/exp OR 'sensitivity and specificity'/de
#12	('predictive value' OR 'predictive values' OR 'detection rate' OR 'detection rates' OR 'False Negative' OR 'False positive' OR 'True Negative' OR 'True positive' OR 'PPV' OR 'NPV' OR Sensitivit* OR Specificit*):ab,ti,kw
#13	(Diagnos* NEAR/3 Accurac*):ab,ti,kw
#14	(Validit*):ab,ti,kw
#15	#11 OR #12 OR #13 OR #14
#16	#4 AND #7 AND #10 AND #15
#17	#16 AND ([english]/lim) AND [2000-2020]/py AND ([article]/lim OR [article in press]/lim OR [review]/lim)

No.	Search queries for Cochrane
#1	[mh "Carcinoma, Hepatocellular"] or [mh ^"Liver Neoplasms"]
#2	((Hepatocellular* or liver-cell* or hepatic-cell* or Hepato-cell*) near/6 (Cancer* or tumor* or Neoplas* or carcinoma*)):ab,ti,kw
#3	hepatocarcinoma*:ab,ti,kw or hepatoma*:ab,ti,kw or "liver carcinoma":ab,ti,kw or HCC:ab,ti,kw
#4	#1 or #2 or #3
#5	[mh "Magnetic Resonance Imaging"]
#6	Magnetic-Resonanc*:ab,ti,kw or "MR":ti or "MRI":ab,ti or "diffusion-weighted":ab,ti,kw
#7	#5 or #6
#8	abbreviat*:ab,ti,kw or surveillanc*:ab,ti,kw or Monitoring*:ab,ti,kw
#9	[mh "Early Detection of Cancer"] or [mh "mass screening"] or [mh "Epidemiological Monitoring"]
#10	#8 or #9
#11	[mh "Predictive Value of Tests"] or [mh "Sensitivity and Specificity"]
#12	(predictive value* or detection rate* or "False Negative" or "False positive" or "True Negative" or "True positive" or "PPV" or "NPV" or Sensitivit* or

	Specificit*):ab,ti,kw
#13	[mh "Reproducibility of Results"]
#14	(Reproducib* or accurac* or Reliabilit* or Validit*):ab,ti,kw
#15	#11 or #12 or #13 or #14
#16	#4 and #7 and #10 and #15
#17	#16 in Trials(Published); 2000-2020

Supplementary Table S2. Performance of abbreviated MRI-protocols and full MRI-protocols for the detection of early-stage HCC and very early-stage HCC

Early-stage HCC					
*Abbreviated MRI-protocols**			*Full MRI-protocols*		
Author (year)	*Sensitivity (95% CI)*	*Specificity (95% CI)*	*Author (year)*	*Sensitivity (95% CI)*	*Specificity (95% CI)*
Sutherland T (2017)	80% (28, 99)	98% (95, 100)	Shah TU (2006)	77% (55, 92)	83% (78, 87)
Brunsing RL (2019)	88% (47, 100)	91% (85, 95)	Kim SY (2017)	86% (71, 94)	97% (96, 98)
			Demirtas CO (2020)	83% (65, 94)	95% (92, 98)
Meta-analytic pooled estimations	85% (66, 100)	96% (91, 100)	Meta-analytic pooled estimations	83% (74, 91)	94% (88, 99)
Very early-stage HCC					
*Abbreviated MRI-protocols**			*Full MRI-protocols*		
Author (year)	*Sensitivity (95% CI)*	*Specificity (95% CI)*	*Author (year)*	*Sensitivity (95% CI)*	*Specificity (95% CI)*
Chan MV (2019)	59% (34, 83)	95% (87, 100)	Shah TU (2006)	77% (55, 92)	83% (78, 87)
			Kim SY (2017)	84% (67, 95)	97% (96, 98)
			Demirtas CO (2020)	80% (52, 96)	96% (93, 98)
Meta-analytic pooled estimations	59% (34, 83)	95% (87, 100)	Meta-analytic pooled estimations	81% (71, 91)	94% (89, 99)

*Consisting of various combinations of imaging sequences without dynamic contrast-enhanced image, including T1-weighted imaging, T2-weighed imaging, diffusion-weighted imaging, and hepatobiliary-phase imaging.

MRI, magnetic resonance imaging; HCC, hepatocellular carcinoma; CI, confidence interval.

Supplementary Table S3. Results of sensitivity analysis of surveillance MRI for the detection of hepatocellular carcinoma

	Sensitivity (95% CI)	I^2	*Specificity (95% CI)*	I^2
Pooled data for seven studies	85% (79, 90)	0%	94% (90, 97)	94%
Excluded study (year)				
Shah TU (2006)	86% (80, 91)	0%	95% (93, 97)	81%
Marks RM (2015)	84% (77, 90)	0%	95% (90, 97)	95%
Kim SY (2017)	85% (78, 90)	0%	93% (88, 96)	92%
Sutherland T (2017)	85% (79, 90)	0%	93% (89, 96)	94%
Brunsing RL (2019)	85% (79, 90)	0%	95% (90, 97)	95%
Chan MV (2019)	86% (79, 90)	0%	94% (89, 97)	95%
Demirtas CO (2020)	85% (79, 90)	0%	94% (89, 97)	94%

MRI, magnetic resonance imaging; CI, confidence interval.

Supplementary Table S4. Results of meta-regression analysis of surveillance MRI for the detection of hepatocellular carcinoma

		Meta-analytic summary estimate		
Covariates	*Subgroup*	*Sensitivity (95% CI)*	*Specificity (95% CI)*	*P-value*
Proportion of HCC < 2 cm	< 50% or NR (n = 4)	86% (79, 93)	95% (92, 99)	0.58
	> 50% (n = 3)	84% (75, 93)	92% (86, 98)	
The most common etiology of underlying liver disease	Hepatitis C virus (n = 3)	85% (77, 93)	88% (84, 92)	0.01
	Hepatitis B virus (n = 4)	85% (78, 92)	97% (95, 98)	
HCC prevalence in each study	< 10% (n = 3)	83% (71, 95)	92% (87, 98)	0.71
	> 10% (n = 4)	86% (80, 91)	95% (92, 98)	
Study location	Western (n = 5)	85% (78, 92)	93% (89, 96)	0.43
	Eastern (n = 2)	86% (78, 94)	96% (93, 99)	
Study period	< 2005 (n = 1)	77% (55, 92)	83% (78, 87)	0.05
	≥ 2005 (n = 6)	86% (81, 91)	95% (93, 97)	
MRI magnet	3.0-T only (n = 2)	84% (75, 93)	95% (90, 100)	0.85
	1.5-T, both 1.5- and 3.0-T, or NR (n = 5)	86% (80, 92)	94% (90, 98)	
MRI contrast agent*	Hepatocyte-specific contrast agent (n = 3)	87% (81, 94)	94% (90, 98)	< 0.01
	Extracellular contrast agent (n = 2)	82% (72, 92)	91% (83, 98)	
Reference standard for HCC	Imaging only (n = 2)	85% (74, 96)	93% (86, 100)	0.92
	Pathology or imaging (n = 5)	85% (80, 91)	94% (91, 98)	
Reference standard for non-HCC	Explantation only (n = 1)	77% (55, 92)	83% (78, 87)	0.05
	Imaging follow-up (n = 6)	86% (81, 91)	95% (93, 97)	
Follow-up period	< mean 6 months (n = 1)	82% (62, 93)	95% (89, 97)	0.87
	≥ mean 6 months (n = 6)	86% (80, 91)	94% (91, 97)	

The results were obtained using meta-regression analysis with the bivariate model.

*Two articles that used non-enhanced MRI were excluded.

MRI, magnetic resonance imaging; HCC, hepatocellular carcinoma; NR, not reported.

Supplementary Figure S1. Deeks' funnel plot to evaluate publication bias of surveillance MRI

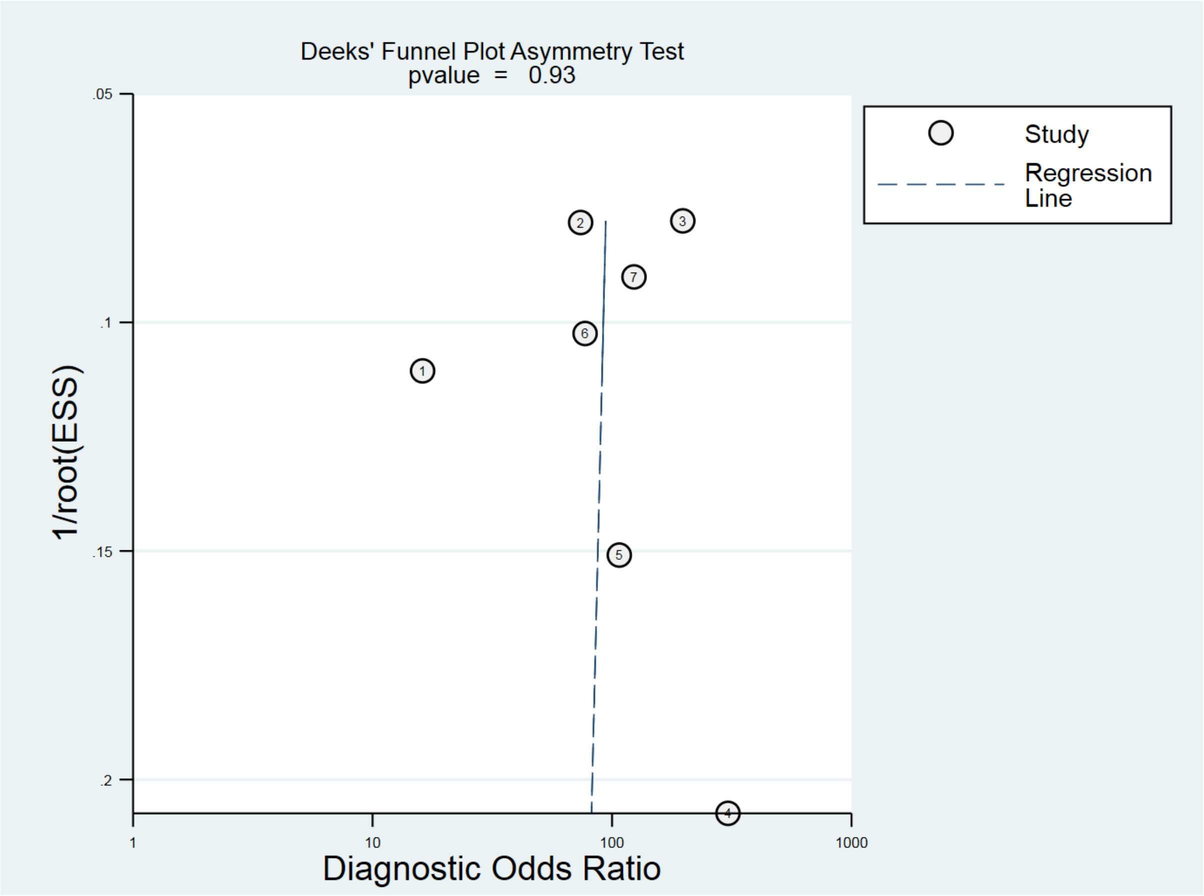

Supplement: Supplementary file 1 [file diagnostics-11-01665-s001.zip › diagnostics-1329652-supplementary.pdf]
